# Local anaesthetics and chemotherapeutic agents: a systematic review of preclinical evidence of interactions and cancer biology

**DOI:** 10.1016/j.bjao.2024.100284

**Published:** 2024-05-05

**Authors:** Ahmed Abdelaatti, Donal J. Buggy, Thomas P. Wall

**Affiliations:** 1 Department of Anaesthesiology & Perioperative Medicine, Mater Misericordiae University Hospital, School of Medicine, University College Dublin, Dublin, Ireland; 2 EuroPeriscope, European Society of Anaesthesiology and Intensive Care - Onco-Anaesthesiology Research Group, Brussels, Belgium; 3 Outcomes Research Consortium, Cleveland Clinic, Cleveland, OH, USA

**Keywords:** anaesthesia, cancer, cancer recurrence, chemotherapy, local anaesthetics

## Abstract

**Background:**

Local anaesthetics are widely used for their analgesic and anaesthetic properties in the perioperative setting, including surgical procedures to excise malignant tumours. Simultaneously, chemotherapeutic agents remain a cornerstone of cancer treatment, targeting rapidly dividing cancer cells to inhibit tumour growth. The potential interactions between these two drug classes have drawn increasing attention and there are oncological surgical contexts where their combined use could be considered. This review examines existing evidence regarding the interactions between local anaesthetics and chemotherapeutic agents, including biological mechanisms and clinical implications.

**Methods:**

A systematic search of electronic databases was performed as per Preferred Reporting Items for Systematic Reviews and Meta-analysis (PRISMA) guidelines. Selection criteria were designed to capture *in vitro*, *in vivo*, and clinical studies assessing interactions between local anaesthetics and a wide variety of chemotherapeutic agents. Screening and data extraction were performed independently by two reviewers. The data were synthesised using a narrative approach because of the anticipated heterogeneity of included studies.

**Results:**

Initial searches yielded 1225 relevant articles for screening, of which 43 met the inclusion criteria. The interactions between local anaesthetics and chemotherapeutic agents were diverse and multifaceted. *In vitro* studies frequently demonstrated altered cytotoxicity profiles when these agents were combined, with variations depending on the specific drug combination and cancer cell type. Mechanistically, some interactions were attributed to modifications in efflux pump activity, tumour suppressor gene expression, or alterations in cellular signalling pathways associated with tumour promotion. A large majority of *in vitro* studies report potentially beneficial effects of local anaesthetics in terms of enhancing the antineoplastic activity of chemotherapeutic agents. In animal models, the combined administration of local anaesthetics and chemotherapeutic agents showed largely beneficial effects on tumour growth, metastasis, and overall survival. Notably, no clinical study examining the possible interactions of local anaesthetics and chemotherapy on cancer outcomes has been reported.

**Conclusions:**

Reported preclinical interactions between local anaesthetics and chemotherapeutic agents are complex and encompass a spectrum of effects which are largely, although not uniformly, additive or synergistic. The clinical implications of these interactions remain unclear because of the lack of prospective trials. Nonetheless, the modulation of chemotherapy effects by local anaesthetics warrants further clinical investigation in the context of cancer surgery where they could be used together.

**Clinical trial registration:**

Open Science Framework (OSF, project link: https://osf.io/r2u4z)

Cancer remains a formidable challenge to global health, representing a leading cause of morbidity and mortality worldwide with almost 10 million cancer-related deaths in 2020.^1^ Oncology is a rapidly evolving field, with significant progress made recently not only with the development of novel therapies, but also with deepening of our understanding of cancer biology and metastasis.^2^ Despite these medical advances, surgery remains the primary curative strategy for solid organ cancers. An area of growing speculation is the potential interaction between local anaesthetics, which are commonly used in perioperative settings for anaesthesia and pain management, and chemotherapeutic agents.

Cancer progression is a highly complex, multifactorial process involving primary tumour growth, release of circulating tumour cells, and the subsequent development of distant metastases.^3^ Metastasis is not only a consequence of the intrinsic characteristics of cancer cells but is also influenced by the host microenvironment and systemic factors.^4^ Research suggests that invasive surgical procedures may have an impact on tumour growth and the likelihood of metastasis.^5^ This raises questions about how the patient's perioperative journey, including choice of drugs administered perioperatively, may influence cancer progression.^6^ Surgical tissue trauma initiates multiple physiological signalling pathways resulting in a complex interplay of adrenergic, inflammatory, and immune responses which are intended to cause wound healing but, inadvertently, are also implicated in stimulating the growth of residual cancer cells. Significant preclinical experimental research has revealed how anaesthetic and analgesic techniques may modulate these pathways and possibly alter the risk of cancer progressing or recurring after operation.^7^ For instance, the use of volatile anaesthetic agents leads to higher postoperative serum pro-inflammatory cytokine concentrations.^8^

Some retrospective clinical studies have detected an association between anaesthetic type and risk of postoperative cancer recurrence, although this association has not uniformly been found.^9^^,^^10^ Although few prospective randomised controlled trials (RCTs) have been completed to date, almost all published RCTs have not confirmed a definite link between technique or agent used and long-term cancer outcomes. For example, there is no high-quality evidence that propofol is superior to volatile agents or that epidural use conveys an advantage in terms of overall survival or cancer recurrence.^11^ The exception is a large RCT involving women undergoing breast cancer surgery of curative intent. Almost 1600 women were randomly allocated to an active arm (received peritumoral infiltration of the local anaesthetic lidocaine 0.5% up to 4.5 mg kg^−1^ body weight, 10 min before surgical excision), compared with a control group, who did not receive local anaesthetic infiltration. In the local anaesthetic and control groups, 5-yr disease-free survival rates were 87% and 83% (hazard ratio [HR] 0.74; 95% confidence interval [CI] 0.58–0.95; *P*=0.017) and 5-yr overall survival rates were 90% and 86%, respectively (HR 0.71; 95% CI 0.53–0.94; *P*=0.019). No toxic effects of lidocaine were observed. Although it has been the subject of some criticism (e.g. for lacking a placebo injection in the control group), this trial is the first to report a positive difference after a single perioperative intervention on long-term oncological outcomes.^12^

Lidocaine and other local anaesthetics have been examined extensively in preclinical studies with substantial laboratory evidence that they can inhibit cancer biology both *in vitro* and *in vivo*.^13^ The mechanisms underlying this effect appear multifactorial with evidence that local anaesthetics have direct inhibitory effects on cancer cells via numerous cellular signalling pathways: they may damage mitochondria directly, they may help to preserve immune cell function, and may inhibit both angiogenesis and inflammation.^14^^,^^15^ These potentially beneficial physiological effects were also detected in clinical translational studies: for instance, i.v. lidocaine infusion has an anti-inflammatory effect and reduces postoperative serum pro-inflammatory cytokine concentrations.^16^

Surgical procedures involving concurrent administration of local anaesthetics and chemotherapeutic agents might at first glance appear limited, but are in fact quite frequent and likely to be steadily increasing. Intraoperative chemotherapy use was previously largely confined to hyperthermic intraperitoneal chemotherapy (HIPEC) using mitomycin C, oxaliplatin, and others, during cytoreductive surgery (CRS) for ovarian malignancies with peritoneal carcinomatosis and pseudomyxoma peritonei, but subsequently this technique was expanded to treat advanced colorectal and gastric malignancies.^17^ In 2018 at least 3800 patients underwent CRS/HIPEC worldwide, although this estimate was based on a limited survey of 19 countries so may have underestimated the true figure.^18^ In some HIPEC cases termed ‘bidirectional HIPEC’, intraoperative i.v. chemotherapy (e.g. i.v. cisplatin) is co-administered with intraperitoneal chemotherapy.^19^ Heated intracavitary chemotherapy combined with CRS is also used in the thoracic cavity for the treatment of malignant pleural mesothelioma.^20^ Infusion of chemotherapeutic agents into the portal vein or hepatic artery using catheters placed during surgery to excise primary colorectal tumours has been studied, with mixed results being reported.^21^^,^^22^ Very recently, some centres have examined whether intraoperative i.v. chemotherapy during colorectal cancer excisional surgery may improve cancer outcomes, with promising initial results.^23^ Intraoperative intravesical chemotherapy has been used during nephroureterectomy to reduce bladder urothelial carcinoma recurrence.^24^ Given the trend in management patterns towards a more interventional approach and a shift in attitudes towards viewing advanced cancers as a chronic illness where multiple, aggressive resections are considered appropriate, it may be expected that procedures involving intraoperative or very early postoperative chemotherapy will increase in frequency.^25^^,^^26^

Looking ahead then, it appears likely that procedures where local anaesthetics and chemotherapeutic agents are co-administered will become more common. Local anaesthetic either given systemically or absorbed into the circulation (from epidural, regional block, infiltration, etc.) may interact with chemotherapeutic drugs similarly given either systemically or absorbed via the peritoneum, pleura or bladder. Local anaesthetics could potentially act to synergistically enhance the anticancer effects of intraoperative chemotherapy, in which case short-term (hours-to-days) exposure to local anaesthetic may potentially improve the patient's long-term survival or cancer recurrence risk. Or local anaesthetic may in fact inhibit chemotherapy effects and should be avoided to reduce the risk of adverse outcomes. Understanding the potential impact of local anaesthetics on cancer recurrence and metastasis is therefore of paramount importance and will allow for optimal perioperative care of the cancer patient to achieve the best long-term outcomes. To the best of our knowledge, this review is the first to evaluate existing literature regarding the interactions between local anaesthetics and chemotherapy.

## Methods

This systematic review was conducted in accordance with the Preferred Reporting Items for Systematic Review and Meta-analysis (PRISMA) guidelines.^27^ The PRISMA checklist can be found in the Supplementary Materials. After initial exploratory scoping searches it was evident that no human clinical studies would be eligible for inclusion and the large majority of included studies would be *in vitro* in nature with a smaller number of *in vivo* studies. Therefore, this review was ineligible for registration in the PROSPERO international prospective register of systematic reviews; instead, the review methodology was predefined and prospectively registered on the Open Science Framework (OSF, project link: https://osf.io/r2u4z), a similar registry including a wider range of review types. The following databases were searched for relevant published literature: PubMed, Scopus, Web of Science and Embase. The search strategy used for each database is available on the OSF registration page. The database searches were performed on 2 August 2023. If any relevant references were found in study bibliographies, then these were included also. In order to be included in the review the returned studies had to meet the following inclusion criteria: (i) preclinical or clinical studies examining cancer; (ii) studies where local anaesthetic agents are combined with a chemotherapy drug (any agent, with common agents specifically identified, see OSF page); (iii) studies where the effect of each drug on its own is compared with both used in combination; (iv) *in vitro* studies which examine outcomes including cell viability, proliferation, migration, protein/gene expression, etc., or clinical outcomes (survival, recurrence, metastasis, tumour sizes, etc.) for any *in vivo* or clinical studies. The exclusion criteria were: (i) studies not published in English; (ii) studies that do not involve combination use of a local anaesthetic and a chemotherapeutic agent (e.g. both are studied but not used in combination). The Covidence online systematic review platform (Veritas Health Innovation, Melbourne, Australia) was used to manage references, identify duplicates, screen studies, and aid with the data extraction process. Studies were screened for eligibility by two independent reviewers (AA, TPW). Studies meeting the inclusion criteria based on their title or abstract, or where their eligibility was uncertain, proceeded to full-text assessment. Each full text was assessed in duplicate by the same reviewers to determine their eligibility for inclusion. Where there was disagreement between the two reviewers as to eligibility, a third reviewer (DJB) made a final decision on inclusion. For eligible references, data were extracted including author, year published, study type (*in vivo* or *in vitro*), cancer type, local anaesthetic(s) examined, local anaesthetic concentration used, chemotherapeutic agent(s) examined, interaction observed and mechanism of action studied (if any). As there were no clinical studies to include, no risk of bias or quality assessment was performed as there are no established methods for performing these assessments for *in vitro* and *in vivo* laboratory studies. Given this limitation, and the anticipated high degree of heterogeneity of the included studies, a narrative synthesis of the extracted data and subsequent findings was performed.

## Results

**Fig 1:**
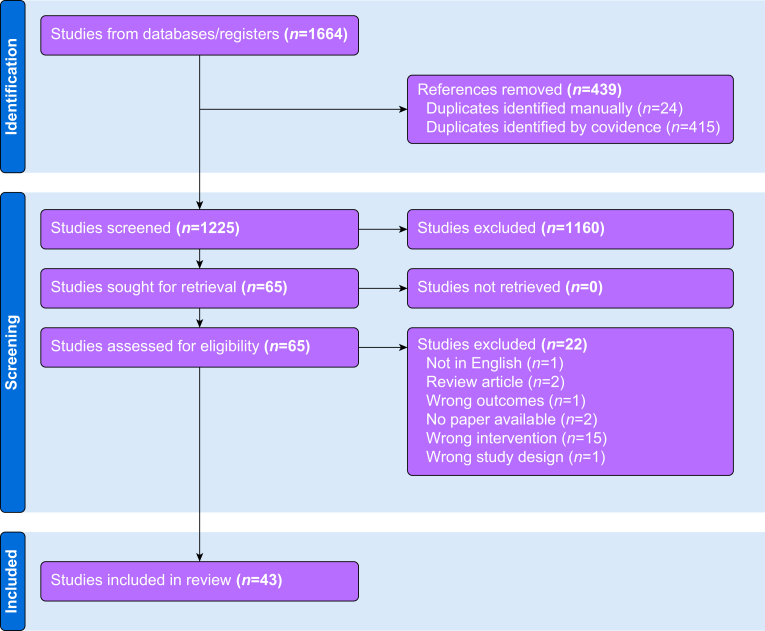
PRISMA flowchart. PRISMA, Preferred Reporting Items for Systematic Reviews and Meta-analysis.

**Fig 2:**
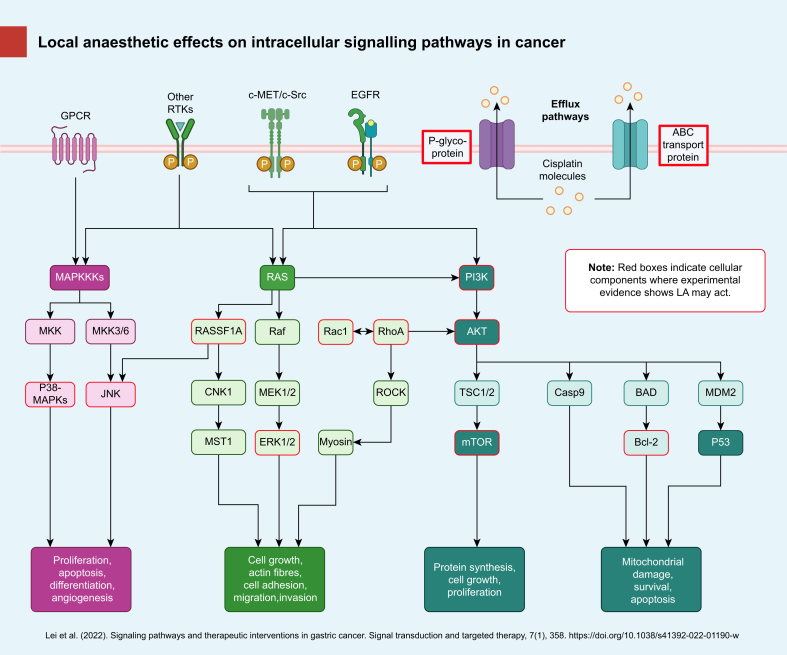
Simplified schematic of selected intracellular signalling pathways in cancer and points at which experimental evidence suggests local anaesthetic agents may act. The basic elements of pathways involving PI3K/AKT/mTOR, MAPK, c-Met, p53, drug efflux mechanisms and the overall cell functions affected are shown. ABC, ATP-binding cassette transporters; AKT, protein kinase B; BAD, Bcl-xl/Bcl-2-associated death promoter; Bcl-2, B-cell lymphoma 2; c-Met, c-mesenchymal-epithelial transition factor; Casp9, cysteinyl aspartate specific proteinase 9; c-Src, Proto-oncogene tyrosine-protein kinase Src; CNK1, connector enhancer of KSR; EGFR, epidermal growth factor receptor; ERK1/2, extracellular signal-related kinase 1/2; GPCRs, G-protein-coupled receptors; JNK, jun amino-terminal kinase; LA, local anaesthetic; MAPKKKs, mitogen-activated protein kinase kinase kinases; MDM2, murine double minute 2; MEK, mitogen-activated protein kinase kinase; MKK, mitogen-activated protein kinase kinase; MST1, mammalian sterile 20 kinase 1; mTOR, mammalian target of rapamycin; p38-MAPKs, p38 group of mitogen-activated protein kinases; p53, tumor protein 53; PI3K, phosphoinositide 3-kinase; PTEN, phosphatase and tensin homolog; Rac1, Ras-related C3 botulinum toxin substrate 1; Raf, rapidly accelerated fibrosarcoma; RAS, rat sarcoma; RASSF1A, Ras association domain-containing protein 1; RhoA, Ras homolog family member A; ROCK, Rho-associated, coiled-coil containing protein kinase 1; RTKs, receptor tyrosine kinases; TSC1/2, tuberous sclerosis complex 1/2. Figure created using Biorender.com.

Figure 1 shows the PRISMA flow diagram for the review process. Initial searches retrieved 1664 references; 439 duplicates were identified and removed leaving 1225 references to be screened by title and abstract. Screening resulted in 65 references that were assessed as full-text articles. After full-text analysis, 43 references were eligible for data extraction. Summary extracted data are presented in Table 1 (studies involving lidocaine and other amino amide local anaesthetics) and Table 2 (studies involving the amino ester procaine). *In vitro* experiments were described in 30 studies, five were *in vivo* animal model experiments and eight involved both *in vitro* and *in vivo* approaches. No clinical study matching the inclusion criteria was identified. Ten different local anaesthetics were examined in preclinical studies: lidocaine, bupivacaine, levobupivacaine, ropivacaine, dibucaine, mepivacaine, procaine, tetracaine, benzocaine, and butacaine. Lidocaine was the most frequently studied local anaesthetic with 27 published articles: 18 involving lidocaine alone and nine studied lidocaine along with other local anaesthetics. Procaine was studied in 17 reports: eight with procaine alone, five where procaine was used as a complex with cisplatin (known as DPR) and four where procaine was examined along with other local anaesthetics. Twenty-three chemotherapeutic agents were analysed across the 42 studies; the most commonly studied were cisplatin (18 studies) and 5-fluorouracil (eight). A hypothetical biochemical mechanism of action was examined in 27 studies, such as deoxyribonucleic acid (DNA) demethylation, P-glycoprotein expression, etc. A total of 16 different cancer types were examined between the 43 included studies, some of which examined more than one type. The most commonly studied were breast cancer (11) and leukaemia (11). The large majority of included studies (38/43, 88.4%) reported potentially beneficial anticancer effects of combined local anaesthetic/chemotherapeutic agent use compared with sole use of either agent. Typical beneficial effects seen *in vitro* were enhanced cytotoxicity or reduced resistance to the chemotherapeutic agent, and typical *in vivo* effects seen were improved survival or reduced tumour size. Three studies did not detect beneficial effects attributable to combination local anaesthetic/chemotherapeutic agent treatment *in vitro* or *in vivo*,^28^, ^29^, ^30^ and two reported potentially harmful effects.^31^^,^^32^ Potential cellular mechanisms of action responsible for the experimental effects observed were investigated by 26 studies, with numerous candidate mechanisms identified ranging from drug efflux protein effects, DNA demethylation, inhibition or promotion of intracellular signalling pathways controlling apoptosis, etc. These putative mechanisms of action are shown schematically in Fig 2.

**Table 1:** Summary extracted data from studies examining amino amide local anaesthetics which meet systematic review selection criteria. 5-FU, 5-fluorouracil; 8-OHdG, 8-hydroxy-2' -deoxyguanosine; ABCG2, ATP-binding cassette G2; AKT, protein kinase B; Bax, BCL2-associated X; Bcl-2, B-cell lymphoma 2; c-Met, c-mesenchymal-epithelial transition factor; c-Src, Proto-oncogene tyrosine-protein kinase Src; DDP, diamminedichloroplatinum; EMT, epithelial-to-mesenchymal transition; ERK1/2, extracellular signal-related kinase 1/2; GSK3B, Glycogen synthase kinase-3 beta; IL-6, interleukin-6; mTOR, mammalian target of rapamycin; miR, micro RNA; MLC, myosin light chain; MMP, matrix-metalloproteinase; MRP, multidrug resistance-associated protein; NLC-DTX, nanostructured lipid carrier-docetaxel; PI3K, phosphoinositide 3-kinase; Rac1, Ras-related C3 botulinum toxin substrate 1; RARβ2, retinoic acid receptor beta 2; RASSF1A, Ras association domain-containing protein 1; ROCK, Rho-associated, coiled-coil containing protein kinase 1; ROS, reactive oxygen species; SCC, squamous cell carcinoma; SIRT, sirtuin 1; SOX4, SRY-related HMG-box 4; TGF, transforming growth factor.

1st author and year	Study type	Cancer type (cell line)	Local anaesthetic(s) studied	Local anaesthetic concentration	Chemotherapy agent(s) studied	Interaction observed (e.g. no effect, synergistic effect, additive effect, etc)	Mechanism(s) studied (if any)
Li 2014^51^	*In vitro*	Breast (MCF-7, MDA-MB-231)	Lidocaine	10 μM–1 mM	Cisplatin	Lidocaine enhanced the cytotoxic effects of cisplatin	Upregulation of RARβ2 and RASSF1A (promoters of tumour suppressor genes)
Xing 2017^46^	Both	Hepatocellular (HepG2)	Lidocaine	100 μM–10 mM *in vitro*, 30 mg kg^−1^ twice weekly *in vivo*	Cisplatin	Lidocaine–cisplatin treatment was more cytotoxic *in vitro* and suppressed tumour growth more effective *in vivo* than either agent used alone	Bcl-2, Bax, and cleaved caspase-3 activation, activation of ERK1/2, p-38 cascade
Yang 2019^49^	*In vitro*	Lung cancer (A549/DDP)	Lidocaine	1–100 μM	Cisplatin	Lidocaine reduced cisplatin resistance	miR-21 expression
Liu 2022^47^	*In vitro*	Skin squamous cell carcinoma (A431)	Lidocaine	0–10 mM	Cisplatin	Lidocaine reduces cisplatin resistance	miR-30c/SIRT1 pathway activation
Freeman 2018^44^	*In vivo*	Breast (4T1 murine)	Lidocaine	1.5 mg kg^−1^ bolus then 2 mg kg^−1^ h^−1^ infusion	Cisplatin	Enhanced effect in terms of reduced pulmonary metastases, no effect on liver metastases	No significant difference in serum IL-6 noted
Zhang 2020^48^	*In vitro*	Gastric (MGC-803, MGC-803/DDP)	Lidocaine	25 μM–200 μM	Cisplatin	Lidocaine reduced cisplatin resistance	Inhibition of miR-10b, AKT/mTOR and β-catenin pathway repression
Gao 2018^45^	Both	Breast (MDA-MB-231, MCF-7)	Lidocaine	*In vitro* 12 mg kg^−1^ (murine model)	Cisplatin	Nanogel loaded lidocaine has a synergistic anticancer effect with co-loaded cisplatin both *in vitro* and *in vivo*	N/A
Lazo 1985^52^	*In vitro*	Leukaemia (L1210 murine)	Lidocaine	0–10 mM	Cisplatin, bleomycin, mitomycin C, etoposide	Lidocaine potentiates bleomycin, cisplatin and etoposide cytotoxicity	N/A
Zeng 2021^43^	*In vitro*	Gastric (MKN45)	Lidocaine	10 mM	Cisplatin, 5-FU	Lidocaine enhanced sensitivity of cells to chemotherapeutic agents	Phosphorylation levels of c-Met and c-Src were reduced by lidocaine treatment
Zhang 2019^40^	*In vitro*	Choriocarcinoma (JEG-3, JAR)	Lidocaine	10–1000 μM	5-FU	Lidocaine potentiated 5-FU cytotoxicity	ATP-binding cassette (ABC) transport protein expression—expression of ABCG2, P-glycoprotein, MRP1, MRP2, PI3K/AKT pathway inhibition
Wang 2017^41^	*In vitro*	Melanoma (SK-MEL-2)	Lidocaine	10–1000 μM	5-FU	Lidocaine enhances sensitivity of melanoma cells to 5-FU	Lidocaine induced expression of miR-493 and downregulated expression of SOX4 perhaps by inactivation of PI3K/AKT and TGF-TGF-β pathways
Polekova 1992^58^	*In vitro*	Leukaemia (L1210, murine)	Lidocaine	0–2 mM	Vincristine	Lidocaine reversed cancer cell resistance to vincristine	P-glycoprotein and MDR1 (multidrug resistance) gene expression
Kim 2019^29^	*In vitro*	Oral SCC (KBV20C, MDR cells)	Lidocaine	5 μM	Vincristine	Lidocaine had no additional effect on cell viability when combined with vincristine	Inhibition of the P-glycoprotein cell efflux protein
Wall 2019^31^	*In vivo*	Breast (4T1 murine)	Lidocaine	1.5 mg kg^−1^ bolus + 2 mg kg^−1^ h^−1^ infusion	Bosutinib	Bosutinib reversed the antimetastatic effect of lidocaine, lidocaine reduced MMP-2 expression	Src, MMP-2/9 inhibition
Wall 2021^30^	*In vitro*	Breast (4T1 murine)	Lidocaine	5 μM–3 mM	Bosutinib	No effect of combination therapy at therapeutic concentrations	N/A
Han 2022^62^	Both	Breast cancer (MDA-MB231 and 453)	Lidocaine	0–3 mM	Palbociclib	Palbociclib effects enhanced by local anaesthetic (*in vivo*/*in vitro*)	Inhibition of PI3K/AKT/GSK3B and EMT signalling
Yang 2018^55^	Both	Bladder (BIU-87)	Lidocaine	1.25–5 mg ml^−1^*in vitro*, 2.5–5 mg ml^−1^*in vivo* per week	Mitomycin C, pirarubicin	*In vitro* lidocaine enhances cytotoxicity of both chemotherapeutic agents, *in vivo* lidocaine/MMC prolonged survival and reduced mean bladder wet weight compared with solo therapy	N/A
De Moura 2021^61^	Both	Melanoma (B16–F10 murine, SK-MEL-103)	Lidocaine	30 μM–10 mM	Docetaxel (DTX)	Addition of lidocaine to NLC-DTX and HGel-NLC-DTX systems increased their cytotoxicity *in vitro*; addition of lidocaine decreased tumour growth *in vivo*	Nanostructured lipid carriers (NLC) combined with the antineoplastic docetaxel, formed a hybrid gel (NLC-in-hydrogel) for topical application
Zheng 2020^56^	*In vitro*	Melanoma (A375, A431)	Lidocaine, ropivacaine, bupivacaine	250 μM–2 mM	Dacarbazine, vemurafenib	Ropivacaine and lidocaine (but not bupivacaine) enhanced the antimigratory, antiproliferative and pro-apoptotic effects of vemurafenib and dacarbazine	Ropivacaine and lidocaine decreased RhoA, Rac1, and Ras activity; bupivacaine did not affect RhoA, Rac1, and Ras activity
Brummelhuis 2021^59^	*In vitro*	Ovarian (OVCAR3, OVCAR5, T47D, KURAMOCHI, JHOS4)	Lidocaine, bupivacaine, benzocaine, procaine	Lidocaine (0.2–125 mM), bupivacaine (6 μM–3.75 mM), benzocaine (8 μM–5 mM), procaine (0.02–12.5 mM)	Carboplatin, paclitaxel	Additive effect of local anaesthetics to chemotherapeutic agent effect	Voltage-gated sodium channel (VGSC) inhibition
Lirk 2014^57^	*In vitro*	Breast (BT-20, MCF-7)	Lidocaine, bupivacaine, ropivacaine	10–309.2 μM	Decitabine (DAC)	No effect of local anaesthetic/DAC combination on cell viability, lidocaine and ropivacaine cause DNA demethylation—this effect is additive (but not supra-additive) when lidocaine is combined with DAC	DNA demethylation
Dorr 1990^28^	*In vitro*	Leukaemia (L-1210, RL-1210)	Lidocaine, procaine	250–350 μM	Mitomycin C	Local anaesthetics did not reverse resistance to mitomycin-C	P-glycoprotein expression
Mizuno 1982^53^	*In vitro*	Breast (FM3A murine)	Lidocaine, procaine, dibucaine, butacaine, tetracaine	0.2 mM–12 mM	Bleomycin	All local anaesthetics enhanced bleomycin cytotoxicity	N/A
Mizuno 1982^54^	*In vitro*	Breast (FM3A, HeLa)	Lidocaine, procaine, dibucaine, butacaine, tetracaine	0–10 mM	Peplomycin	All local anaesthetics enhanced the cytotoxicity of peplomycin, this was enhanced further by hyperthermia	N/A
Chen 2020^50^	*In vitro*	Hepatoma (HepG2, BEL-7402)	Lidocaine, ropivacaine, bupivacaine	0.05, 0.5, 5 mM	Cisplatin	Chemotherapeutic effect enhanced by local anaesthetics	Unregulated RASSF1A expression
Zhu 2020^42^	*In vitro*	Oesophageal (OE19, SK-GT-4)	Lidocaine, ropivacaine, bupivacaine, mepivacaine	10–100 μM	5-FU, paclitaxel	Local anaesthetics augmented the effects of chemotherapeutic agent drugs in inhibiting growth and inducing apoptosis	Mitochondrial dysfunction and oxidative damage (decreased oxygen consumption rate, increased intercellular ROS and 8-OHdG levels), decreased Rac1 activity, no effect on RhoA
Meireles 2018^60^	*In vitro*	Prostate (PC3)	Lidocaine, ropivacaine, levobupivacaine	Lidocaine (426.7–853.4 nM), ropivacaine (36.4–273.3 nM), levobupivacaine (43.3–173.4 nM)	Docetaxel	Local anaesthetics enhanced chemo-induced inhibition of cell proliferation	N/A
Zheng 2018^66^	*In vitro*	Leukaemia (CD34, K562, LAMA84)	Ropivacaine	100–1000 μM	Dasatinib, imatinib	Local anaesthetic/chemotherapeutic agent combination causes greater growth inhibition and apoptosis induction than either agent used alone	PI3K/Akt/mTOR pathway inhibition, increased caspase-3 activation
Gong 2018^65^	*In vitro*	Breast (MDA-MB-468, SkBr)	Ropivacaine	0.1–1 mM	5-FU	Enhanced effects of 5-FU on inhibiting cell growth, survival, and colony formation	Inhibition of mitochondrial respiration (inhibition of phosphorylation of Akt, mTOR, rS6, and EBP1)
Dan 2018^67^	*In vitro*	Gastric cancer (SNU1, AGS)	Bupivacaine	10 μM–5 mM	5-FU	Inhibitory chemotherapeutic effects augmented by bupivacaine	Inhibition of RhoA/ROCK/MLC

**Table 2:** Summary extracted data from preclinical studies examining the amino ester local anaesthetic procaine which meet systematic review selection criteria. 5-FU, 5-fluorouracil; DDP, diamminedichloroplatinum; DPR, cis-diamminechloro-[2-(diethylamino)ethyl 4-amino-benzoate, N4]-chlorideplatinum(II); PAX9, Paired box gene 9.

1st author and year	Study type	Cancer type (cell line)	Local anaesthetic(s) studied	Local anaesthetic concentration	Chemotherapy agent(s) studied	Interaction observed (e.g. no effect, synergistic effect, additive effect, etc)	Mechanism(s) studied (if any)
Bhol 2022^72^	Both	Oral squamous cell carcinoma (OSCC) (CAL33, FaDu)	Procaine	0.1–1 mM	*In vitro*—cisplatin, 5-FU, doxorubicin, docetaxel; *in vivo*—cisplatin	Procaine enhanced the *in vitro* chemotherapeutic sensitivity of all drugs tested when used in combination; *in vivo*, combination procaine/cisplatin reduced tumour volume compared with single agent	Procaine suppresses DNA methyltransferase and increases the expression of PAX9, a transcription factor downregulated in OSCC
Esposito 1990^76^	Both	Leukaemia (P388 murine, CCRF-CEM, K-562)	Procaine	1–100 mM *in vitro*, 20–100 mg kg^−1^*in vivo*	Cisplatin	No effect on cytotoxicity when procaine added to cisplatin *in vitro*, procaine increased tolerated dose of cisplatin in mice with reduction in nephrotoxicity	N/A
Esposito 1996^77^	*In vivo*	Leukaemia (P388 murine)	Procaine	40 mg kg^−1^	Cisplatin	*In vivo* section examined cisplatin *vs* cisplatin + procaine and cisplatin + procainamide; combined local anaesthetic agent/chemotherapeutic agent improved survival *vs* cisplatin alone	N/A
Viale 2001^78^	*In vivo*	Leukaemia (P388 murine)	Procaine	40 mg kg^−1^	Cisplatin	Procaine–cisplatin increased survival and cure rate compared with cisplatin treatment alone	N/A
Viale 1994^79^	*In vitro*	Leukaemia (P388 murine)	Procaine (as DPR with cisplatin)	0–33.2 μM DPR	Cisplatin	Similar activity of DPR and cisplatin observed	N/A
Viale 1996^83^	Both	Leukaemia (P388, L1210—murine)	Procaine (as DPR with cisplatin)	0.025–1.04 μM DPR *in vitro*; 0.5–10 mg kg^−1^ DPR *in vivo*	Cisplatin	*In vitro*: synergistic effects of combined DDP/DPR on cell survival compared to either agent alone. *In vivo*: DPR/DDP combo synergistically reduced tumour size and increased tumour free mice	N/A
Mariggio 2004^81^	*In vitro*	Multiple types including neuroblastoma, leukaemia, lung cancer	Procaine (as DPR with cisplatin)	Varied, ranges from 0.07 plus or minus 0.02 μM to 48.3 plus or minus 14.9 μM DPR	Cisplatin	Procaine–cisplatin complex (as DPR) causes greater inhibition of cell viability and enhances apoptosis in some cancer cell lines compared with cisplatin alone	DNA fragmentation, DNA inter-strand cross-linking, p53 upregulation by DPR
Viale 2003^80^	*In vitro*	Leukaemia (HL60, K562, Molt-4)	Procaine (as DPR with cisplatin)	0.01–100 μM	Cisplatin, carboplatin	Procaine–cisplatin (as DPR) inhibits colony formation more than cisplatin or carboplatin alone	N/A
Viale 1998^82^	*In vitro*	Ovarian (M5076—murine, A2780)	Procaine (as DPR with cisplatin)	DPR 0.13–4.15 μM M5076, 0.065–1.04 μM A2780	Cisplatin, 5-FU, doxorubicin, mitomycin-c, Taxol	Synergistic or additive effects observed for most DPR–other agent combinations in terms of cytotoxicity	N/A
Ali 2018^73^	*In vitro*	Breast cancer (MCF-7)	Procaine	5–50 μM	Doxorubicin	Procaine enhanced the cytotoxic and apoptotic effect of doxorubicin	DNA binding by procaine
Sabit 2016^32^	*In vitro*	Colon (HCT116)	Procaine	3–5 μM	Carboplatin, erlotinib, vorinostat, Na phenylbutyrate	Procaine and low-dose carboplatin effectively reduced DNA methylation, but higher doses of some drugs increased cell proliferation and methylation	DNA fragmentation
Godal 1969^74^	*In vivo*	Sarcoma	Procaine	4 mg kg^−1^ day^−1^	Nitrogen mustard (HN3)	Procaine co-treatment increased survival	Vasodilation induced by procaine
Chlebowski 1982^75^	*In vitro*	Melanoma (SHG)	Procaine	0.64 mg ml^−1^	Doxorubicin	Combination treatment caused greater cytotoxic effects than single use of either agent	N/A

## Discussion

### Local anaesthetic toxicity and experimental concentrations

When evaluating experimental use of local anaesthetics, care must be taken to consider the concentrations being examined. Local anaesthetic systemic toxicity is an obvious concern with any local anaesthetic and clinicians must ensure that doses do not exceed known safe maximum limits e.g. bupivacaine 2 mg kg^−1^ given as a peripheral nerve block should maintain peak plasma concentration below the toxic concentration for bupivacaine of 1–2 μg ml^−1^.^33^ Although systemic absorption of submaximal doses of local anaesthetic from the epidural space, peripheral nerve block or tissue infiltration will result in low circulating plasma concentrations below the toxic threshold, the local tissue concentrations of local anaesthetic at the site of injection are much higher and may be temporarily as high as the molar concentration of the undiluted injectate (e.g. bupivacaine 0.5% has a molarity of 15.4 mM, the molarity of lidocaine 2% is 85.3 mM). Many of the *in vitro* studies included in this review used molar local anaesthetic concentrations that would be toxic if they reflected plasma concentrations in a human, which would limit the applicability of their results when considering i.v. lidocaine or systemic absorption from neuraxial or regional techniques. However, such concentrations may be safely achievable locally in tissue if local anaesthetic is administered as peritumoral infiltration (as performed with lidocaine in the recently reported breast cancer trial by Badwe and colleagues^12^) or by direct application to a surface (e.g. peritoneum). The 13 *in vivo* local anaesthetic/chemotherapeutic agent experiments included in the review are particularly useful then, because not only are animal models more translatable to human physiology, but *in vivo* studies are also largely restricted to using non-toxic doses mirroring what could safely be used in clinical practice.

### Amino amide local anaesthetics—lidocaine

Lidocaine is the prototype amino amide local anaesthetic and is in widespread use worldwide, appearing on the World Health Organisation's List of Essential Medicines (alongside bupivacaine).^34^ With a short half-life and relatively low toxicity, lidocaine is the only amino amide local anaesthetic which can safely be administered as an i.v. infusion and toxicity is not typically encountered below plasma concentrations of 5 μg ml^−1^ (approximately 22μM).^14^ I.V. lidocaine (typically given as a bolus of 1–2 mg kg^−1^ followed by an infusion of 1–2 mg kg^−1^ h^−1^) has been advocated as a perioperative analgesic.^35^ Only one large RCT has examined perioperative i.v. lidocaine and cancer outcomes, with no effect on overall survival or disease-free survival detected in patients undergoing pancreatectomy for pancreatic cancer, despite a significant survival benefit being detected in a prior large retrospective study.^36^^,^^37^ However, pancreatic cancer is notoriously aggressive, with an almost uniformly dismal prognosis, so how generalisable this result is to other cancers is unclear—other prospective trials examining perioperative i.v. lidocaine are underway or planned.^38^^,^^39^ Otherwise, lidocaine may be administered via the epidural route, used in regional nerve blocks or given as local infiltration (where its use has previously been associated with improved cancer outcomes after surgery for early breast cancer).^12^

### Lidocaine and 5-fluorouracil

Four *in vitro* studies examined lidocaine and 5-fluorouracil used in combination and all demonstrated beneficial antineoplastic effects associated with combination treatment. Zhang and colleagues^40^ studied combination effects on choriocarcinoma cells and found that lidocaine in low concentrations on its own had no effect on cell viability or apoptosis, but at the same concentrations it potentiated 5-fluorouracil cytotoxicity. Mechanistically, this was associated with reduced expression of the ABC efflux protein and inhibition of the phosphoinositide 3-kinase/protein kinase B/mammalian target of rapamycin (PI3K/AKT/mTOR) pathway, a vital cellular signalling pathway that contributes to cell cycle regulation, and increased expression of which is associated with cancer progression and chemotherapy resistance. Inhibition of the PI3K/AKT/mTOR pathway was also found by another group studying lidocaine and 5-fluorouracil effects on melanoma cells. This effect may be mediated by increased expression of the microRNA (miRNA) miR-493, inactivating PI3K/AKT and transforming growth factor- ß (TGF-ß) leading to downregulated expression of SOX4, a transcription factor involved in cell differentiation and proliferation which is often aberrantly expressed in malignancies.^41^ One study examining four amino amide local anaesthetics (lidocaine, ropivacaine, mepivacaine, bupivacaine) found that all substantially augmented the inhibitory effects of 5-fluorouracil in oesophageal cancer cells; this effect may be mediated through inhibition of mitochondrial respiration and Ras-related C3 botulinum toxin substrate 1 (Rac1), a member of the Ras family of GTPases which act as molecular switches to control cytoskeletal rearrangement and dysfunction of which is implicated in oncogenesis.^42^ Lidocaine also enhanced the *in vitro* effect of 5-fluorouracil in a study examining gastric cancer cells, an effect that may be associated with lidocaine-related inhibition of the c-mesenchymal-epithelial transition factor (c-Met)/Proto-oncogene tyrosine-protein kinase Src (c-Src) pathways leading to decreased B-cell lymphoma 2 (Bcl-2) levels and increased levels of Bax and cleaved caspase-3, resulting in apoptosis.^43^

### Lidocaine and cisplatin

Ten studies examined the interaction between lidocaine and cisplatin on cancer biology: seven were *in vitro* studies, one solely *in vivo*, and two combined both approaches. All 10 studies reported some form of beneficial antineoplastic effect when combination therapy was used compared with either drug used alone. Freeman and colleagues^44^ randomly allocated mice with breast cancer to receive either cisplatin, lidocaine, or both, or neither (control) while undergoing resection of the primary tumour under sevoflurane anaesthesia. The pulmonary metastatic burden was measured 14 days after operation; cisplatin reduced lung metastases compared with control, and the addition of lidocaine to cisplatin enhanced this effect. Gao and colleagues^45^ developed a nanogel to deliver cisplatin directly at the site of breast cancer and found that the introduction of lidocaine to the gel not only increased cisplatin-induced apoptosis both *in vitro* and in a mouse model of breast cancer, but also reduced metastasis in the animal model. Additionally, another group developed a murine model of hepatocellular carcinoma and examined lidocaine and cisplatin treatment *in vitro* and *in vivo*. Again, combination lidocaine–cisplatin treatment was significantly more cytotoxic *in vitro* and suppressed tumour growth more effectively *in vivo* than either agent used alone.^46^ These findings were associated with an increased ratio of Bax/Bcl-2 and cleaved caspase-3 activation and activation of the extracellular signal-related kinase 1/2 (ERK1/2) and p38 mitogen-activated protein kinase (MAPK) signalling pathways, suggesting that lidocaine induces apoptosis through these mechanisms.

The seven *in vitro* studies involved a variety of cancer types. The addition of lidocaine potentiated the effects of cisplatin in each of these experiments. A range of putative cellular mechanisms which may explain this interaction were examined. Lidocaine-induced alteration of miRNA expression was examined as a potential mechanism. The miRNAs are small, non-coding RNAs which influence gene expression by binding to target messenger RNA (mRNA) and inhibiting translation. Dysregulated miRNA expression by cancer cells can instigate phenotypical changes in cells within the tumour microenvironment, promoting tumorigenesis and chemotherapy resistance by targeting oncogenes. Three studies found that individual miRNA expression was altered by lidocaine leading to an effect on oncogenic cellular signalling pathways influencing cancer progression—miR30c and sirtuin 1 (SIRT1) in cutaneous squamous cell carcinoma, miR-10b and AKT/mTOR and β-catenin in gastric cancer, and miR-21 and phosphatase and tensin homolog (PTEN)/PI3K/AKT and programmed cell death 4 (PDCD4)/jun amino-terminal kinase (JNK) in lung cancer.^47^, ^48^, ^49^ Two other studies identified upregulated expression of the tumour suppressor gene Ras association domain family 1A (*RASSF1A*) as a potential mechanism for the observed effect of lidocaine potentiating the cytotoxicity of cisplatin on breast cancer and hepatocellular carcinoma cells *in vitro*.^50^^,^^51^

### Lidocaine and other chemotherapeutic agents

Fourteen studies examined the effects of lidocaine and other (non-5-fluorouracil, non-cisplatin) chemotherapeutic agents (one solely *in vivo*, 10 *in vitro*, and three with both *in vivo* and *in vitro* experiments). These involved a wide range of chemotherapeutic agents including cytotoxic antibiotics (mitomycin-C,^28^ bleomycin,^52^^,^^53^ peplomycin^54^), anthracyclines (pirarubicin^55^), other alkylating agents (dacarbazine^56^), antimetabolites (decitabine^57^), vinca alkaloids (vincristine^29^^,^^58^), taxanes (paclitaxel,^59^ docetaxel^60^^,^^61^), cyclin-dependent kinase inhibitors (palbociclib^62^), and Src/BCR-ABL inhibitors (bosutinib^30^^,^^31^). A wide range of cancers were studied but breast cancer was the most common (6/14). Nine studies found that lidocaine potentiated the antineoplastic effects of the chemotherapeutic agent when tested *in vitro*. Of note, four studies did not detect any alteration of chemotherapeutic agent effects by the tested local anaesthetic during combination treatment. Three studies examined P-glycoprotein expression, activity, or both as a potential mechanism by which lidocaine may interact with chemotherapeutic agents.^28^^,^^29^^,^^58^ P-glycoprotein facilitates drug efflux across the cell membrane and out of the cancer cell, thus providing a means by which drug resistance can develop and which provides a site where local anaesthetics can act to reduce P-glycoprotein activity and enhance chemotherapeutic agent efficacy.^63^

One *in vivo* study examining whether lidocaine may act via inhibition of the Src oncogene studied the combination of i.v. lidocaine and bosutinib (a BCR-ABL, Src tyrosine kinase inhibitor) on metastatic outcomes after excision of primary tumours in a mouse model of breast cancer.^31^ While bosutinib alone had no effect on postoperative pulmonary metastases, lidocaine on its own reduced metastases, and unusually, the combination of bosutinib/lidocaine actually reversed the beneficial antimetastatic effects seen with lidocaine alone. A number of explanations were postulated for this phenomenon, including competitive binding at receptor sites. In contrast to the results of this study, three other experiments found that lidocaine significantly enhanced the effects of the chemotherapeutic agent being studied when given as combined treatment in murine cancer models: docetaxel in melanoma, mitomycin-C in bladder cancer, and palbociclib in breast cancer.^55^^,^^61^^,^^62^ The beneficial effects attributed to combination local anaesthetic/chemotherapeutic agent treatment in these animal models included reduced tumour growth^61^^,^^62^ and increased survival.^55^

### Other amino amide local anaesthetics

Eleven of the included studies examined non-lidocaine amide local anaesthetics, with individual studies often examining multiple local anaesthetics. The most frequently examined were ropivacaine (7/11) and bupivacaine (6/11), two agents used in everyday clinical practice. All 11 were *in vitro* experiments. Again, a wide range of cancers and chemotherapeutic agents were studied, the most frequent being breast cancer (4/11) and 5-fluorouracil (3/11), respectively. The 11 studies reported almost uniformly beneficial anticancer *in vitro* effects when local anaesthetic was added to chemotherapeutic agent with the inhibitory effects of the chemotherapeutic agent being augmented by those of the local anaesthetic during combination use. The influence of local anaesthetic/chemotherapeutic agent on the Ras and Ras homolog family member A (RhoA)/Rac1 signalling pathways were assessed by three studies. *Ras* is an oncogene coding for a GTPase which stimulates cellular proliferation and survival through, amongst others, the PI3K/AKT/mTOR pathway, and is frequently found in mutated form in cancer and was examined by several included studies.^64^, ^65^, ^66^ RhoA and Rac1 are also GTPases and control actin fibre dynamics, cellular adhesion, and cell migration. One study found that in melanoma cells, lidocaine and ropivacaine, but not bupivacaine, decreased Ras, RhoA, and Rac1 expression, and augmented the antimigratory, antiproliferative and pro-apoptotic effects of the chemotherapeutic agents vemurafenib and dacarbazine when used in combination.^56^ Despite the lack of effect of bupivacaine on these GTPases observed in this melanoma study, another study found that bupivacaine did have inhibitory effects on RhoA and Rac1 in gastric cancer cells; this finding was associated with inhibition of migration and was additive when combined with 5-fluorouracil.^67^ A further study examining lidocaine, bupivacaine, ropivacaine, and mepivacaine found that all tested local anaesthetics significantly enhanced the antiproliferative and pro-apoptotic effects of both 5-fluorouracil and paclitaxel in oesophageal cancer cells, and although RhoA was unaffected, Rac1 activity was significantly depressed by ropivacaine and bupivacaine.^42^ Bupivacaine appeared to be the only local anaesthetic tested that inhibited cell migration at clinically relevant concentrations (10 μM).

### Amino ester local anaesthetic agents

Seventeen studies examined the effects of combined treatment with procaine and a chemotherapeutic agent (11 were *in vitro* studies, three *in vivo*, and three combined both *in vitro* and *in vivo* elements). Procaine (often known by its trade name Novocain) is one of the oldest synthetic local anaesthetics, and although its systemic toxicity is relatively low, it has a high pKa and a low lipid solubility conferring a slow onset time and short duration of action limiting its clinical utility. Historically, i.v. procaine has been used as a sole agent to provide general anaesthesia, as when given in sufficient doses (1.25–2.5 g over 10 min) it will induce reduced consciousness without respiratory depression or cardiovascular collapse. Unsurprisingly, the overall patient experience was frequently unpleasant.^68^ Although still used for dental anaesthesia, the clinical use of procaine has largely been supplanted by newer amide local anaesthetics which are less allergenic and have more favourable onset and offset profiles. However, a closely related derivative, chloroprocaine, has found a useful niche as a short-acting agent for spinal anaesthesia in day case surgery.

Procaine has activity outside of voltage-gated sodium channel blockade with antagonistic effects at acetylcholine receptors, *N*-methyl-d-aspartate (NMDA) receptors, the serotonin receptor-ion channel complex, amongst others.^69^ One potentially beneficial antineoplastic effect of procaine is its recognised ability to demethylate DNA.^70^ DNA methylation is an epigenetic modification involved in regulation of gene expression and when occurring abnormally may result in dysregulated gene expression leading to malignant transformation. DNA hypermethylation resulting in the silencing of tumour suppressor genes is a hallmark of many cancers, and DNA demethylating agents such as 5-aza-2′-deoxycytidine (decitabine) can be effective chemotherapeutic drugs.^71^ Recent evidence has shown that in oral squamous cell carcinoma, procaine inhibits DNA methyltransferase, thereby preventing DNA methylation and promoting the expression of the tumour suppressor gene *PAX9*, eventually leading to inhibition of cell growth and stimulation of apoptosis *in vitro* and *in vivo*.^72^ The effect of procaine on DNA methylation was also identified when used in conjunction with carboplatin in colon cancer cells, leading to inhibition of cancer cell proliferation, although interestingly this effect was abolished at higher doses.^32^ Procaine may also affect DNA by binding directly to certain regions within the double helix, a finding associated with enhanced doxorubicin cytotoxicity when used in combination in breast cancer cells.^73^ The ability of procaine to act as a DNA demethylating agent or otherwise interact with DNA opens a potential door to its use in cancer therapeutics, particularly if additive or synergistic effects with a chemotherapeutic agent can be achieved clinically.

Historically the beneficial antineoplastic effects of procaine were recognised from as early as the 1960s and developed further over the subsequent decades. In 1969 it was observed that procaine enhanced survival in a rat model of sarcoma when combined with nitrogen mustard (an early chemotherapeutic agent) compared with nitrogen mustard given alone, whereas procaine alone had no beneficial effect on survival. This effect was attributed to vasodilatation induced by procaine improving perfusion of the sarcoma by the chemotherapeutic agent.^74^ In the early 1980s, researchers found that procaine enhanced the cytotoxicity of bleomycin (and its derivative peplomycin) to breast cancer cells and doxorubicin to melanoma cells *in vitro*.^53^^,^^54^^,^^75^ Beginning in the early 1990s, Esposito and colleagues^76^^,^^77^ and Viale and colleagues^78^ began experiments examining procaine used along with cisplatin in a murine model of leukaemia, finding that procaine increased the tolerated dose of cisplatin, reduced nephrotoxicity, and improved survival. The same group subsequently developed a novel single molecule combination of procaine and cisplatin known as DPR.^79^^,^^80^ This was tested across a range of cancer types in both *in vitro* and *in vivo* settings and found to have greater antineoplastic effects compared with cisplatin use alone.^81^, ^82^, ^83^ Despite these promising findings, DPR has not found its way into mainstream clinical use as a chemotherapy agent. Esters other than procaine (e.g. benzocaine, butacaine, tetracaine) have rarely been studied in combination with chemotherapeutic agents, but have been associated with beneficial additive effects in the few *in vitro* studies that have been performed.^54^^,^^84^

### Limitations

As with any review, the accuracy of the conclusions we can infer from the evidence is limited by the number, nature, and quality of the included studies themselves. Laboratory studies, unlike clinical trials, lack a recognised framework with which to systematically assess bias, such as the Cochrane Risk of Bias 2 tool (RoB2). Similarly, there are no consensus-based tools with which to examine the quality of the evidence provided by *in vitro* and *in vivo* experiments in a systematic manner as there are for clinical studies (i.e. the GRADE system, Grading of Recommendations, Assessment, Development and Evaluation). The studies included in this review are heterogenous in terms of the local anaesthetics, chemotherapeutic agents, drug concentrations, cancer types, animal models, and outcomes studied. This renders formal meta-analysis inappropriate, and therefore interpretation and synthesis of the results requires a less structured and defined narrative approach, providing a more qualitative rather than quantitative overview of the published evidence. Publication bias favouring studies with positive results may be present also, and it may be that other laboratory studies with negative results were performed but were not published (or were rejected for publication).

### Conclusions

Despite a relatively small evidence base of 43 published preclinical studies, a large majority (almost 90%) of these demonstrated beneficial interactions between local anaesthetics and chemotherapeutic agents in terms of effects on cancer biology across a broad spectrum of cancers. Reassuringly, many of the beneficial *in vitro* effects reported were also detected in animal models, with no evidence of systemic toxicity reported when using conventional doses. Positive results with a good safety profile in animal models present a promising signal that similar results may potentially be seen with translation to human clinical studies. However, no such study has been performed to date. Although the local anaesthetic concentrations used in many of the *in vitro* experiments often exceeded safe plasma concentrations and therefore would not translate to use of i.v. local anaesthetic, these concentrations could be achieved clinically through either local infiltration, topical or intra-cavity application. Perhaps the most obvious cohort in whom interactions between local anaesthetic and chemotherapeutic agent therapy could be studied are those patients undergoing HIPEC where the chemotherapy given into the peritoneal space could possibly interact with, for example, lidocaine given i.v., bupivacaine given via the epidural space and absorbed systemically, or ropivacaine given directly into the peritoneal cavity. As the use of intraoperative chemotherapy becomes more common and broadens in scope, the opportunities for combined local anaesthetic/chemotherapeutic agent use are likely to increase. Whether such combined use may benefit patients in terms of improved clinical cancer outcomes will remain unknown until the completion of suitably powered randomised clinical studies.

## Authors’ contributions

Writing, review, and approval of the final version of the manuscript: all authors.

## Declarations of interest

The authors declare that they have no conflicts of interest.
